# Children’s voices on their values and moral dilemmas when being cared and treated for cancer– a qualitative interview study

**DOI:** 10.1186/s12910-024-01075-3

**Published:** 2024-06-26

**Authors:** Charlotte Weiner, Pernilla Pergert, Anders Castor, Bert Molewijk, Cecilia Bartholdson

**Affiliations:** 1 Childhood Cancer Research Unit, Department of Women’s & Children’s Health, Karolinska Institutet, Stockholm, Sweden; 2 Children’s Regional Health Care, Astrid Lindgren Children’s Hospital, Karolinska University Hospital, Stockholm, Sweden; 3 Department of Public Health and Caring Sciences, Centre for Research Ethics & Bioethics (CRB), Uppsala University, Uppsala, Sweden; 4 Department of Paediatrics, Lund University, Lund, Sweden; 5 Department of Ethics, Law and Medical Humanities, Amsterdam University Medical Center, Amsterdam, Netherlands; 6 Center for Medical Ethics, University of Oslo, Oslo, Norway; 7 Paediatric Neurology and Musculoskeletal Disorders and Homecare, Astrid Lindgren Children’s Hospital, Karolinska University Hospital, Stockholm, Sweden

**Keywords:** Childhood cancer care, Children, Ethics, Moral dilemmas, Values, Qualitative

## Abstract

**Background:**

Childhood cancers affect about 350 children every year in Sweden and are life-threatening diseases. During the treatment period, situations arise that can become morally challenging for the child. When knowing children’s values and morally challenging situations in childhood cancer care, targeted ethics support could be developed and used in care.

**Aim:**

To explore children’s values and moral dilemmas ​​when undergoing cancer treatment.

**Methods:**

This is a qualitative study based on empirical data. The data collection was conducted through three focus group interviews and six individual interviews with children between 10 and 18 years (*n* = 16). A content analysis methodology was used to generate themes. Children who were/have been treated for cancer at three childhood cancer centres in Sweden were invited to participate. The study was approved by the Swedish Ethical Review Authority. The children’s participation was based on voluntariness and consent/assent.

**Findings:**

During the analysis, five themes of values emerged: *Personal relationships, Bodily ease and identity, Feeling in control and being involved, Positive distractions* and *Right care that is needed.* Their moral dilemmas were thematized into: *Should I consider others or not? Should I rest or not?* and *Should I refuse treatment or not?*

**Conclusion:**

Children undergoing cancer treatment want to have personal relationships with healthcare professionals. Their moral dilemmas were about questioning their own physical and psychological well-being against their expectations, the values of others and the treatment required. Further research is needed to understand how to deal with moral dilemmas in children undergoing cancer treatment.

**Supplementary Information:**

The online version contains supplementary material available at 10.1186/s12910-024-01075-3.

## Background

Childhood cancers are life-threatening diseases that affects approximately 350 children each year in Sweden [1]. With modern risk-adapted treatments, more than 80% of children with cancer are cured in high-income countries [2]. However, in Sweden it is still the most common cause of death among children aged 1–14 [3]. Despite the intensive treatments, there is still a significant mortality and morbidity to childhood cancers, which means that the children and their families must endure not only complicated treatments and procedures but also anxieties and fears [4]. Furthermore, children and families are exposed to making decisions about participating in clinical trials and various research projects [5, 6].

Previous research highlights that cancer treatment leads not only to physical consequences but also various psychosocial consequences, which for some children affects their social life for several years. For example, Swedish studies show that some children struggle with their changes in identity and social life [7], as well as feelings of powerlessness, and being alienated and isolated after receiving their cancer diagnosis [8]. According to Kim and Lee [9], those symptoms of social disturbance, including family problems, caused psychological stress which continued a long time after treatment.

When a child is treated for cancer, sometimes moral dilemmas arise for those involved, i.e., the child, the parents and healthcare professionals (HCPs). Moral dilemmas are often grounded in intrapersonal and interpersonal value conflicts about what is morally right to do and for whose sake [10], including questions such as: What is important? and What should one do? [11]

When facing moral dilemmas, individuals are forced to decide between competing values, and the outcome of their decision involves consequences, including harm/loss [12]. Moral dilemmas in childhood cancer care often entail uncertainty of what is best for the child and the possible impact on the child´s care. Previous research in childhood cancer care reveals that moral dilemmas from HCPs’ perspectives are related to decision making with parents [13–16], the child’s growing autonomy [17, 18], treatment limitations [18, 19], and responsibilities of care [19]. Little is known about parents’ moral dilemmas. One study reveals that mothers struggle with treatment decisions that could be life-threatening for their child, for example, deciding whether the child should participate in medical trials or not [20]. In a systematic review from 2018 authors present that most parents felt they would disappoint their doctor if they refused to participate in the trial [21]. A recent study show that parents experienced moral dilemmas when choosing whether to endure the suffering of their child passively or to take action to stop it [22]. Even though children represent a core perspective in the triad of childhood cancer care, research on moral dilemmas from the perspective of children is scarce. There are some studies on children’s general experiences in childhood cancer care that include their obstacles in decision-making situations [6], fertility preservation [23, 24], and decisions regarding end-of-life questions [25], yet we lack a specific focus on children’s moral values and dilemmas.

In previous research, authors have argued that there is a need for HCPs to increase their knowledge about what children experience when they undergo cancer treatment [7], which could include insights into children’s values ​​and moral dilemmas. Furthermore, there is a need for knowledge about how HCPs should meet and talk about children’s experiences [26], as this will better help HCPs to offer good and qualified care. The child’s values ​​and moral dilemmas can become an important component in being able to understand the triad of perspectives in difficult situations within Swedish childhood cancer care. Moreover, it is important to explore moral dilemmas from children’s perspectives enabling consideration of their voice and meeting the child’s right to participate in their care [27].

## Aim

To explore children’s values and moral dilemmas ​​when undergoing childhood cancer treatment.

## Methods

### Study design and participants

This descriptive explorative qualitative study was conducted among children who were being/had been treated for childhood cancer. Data from individual interviews and focus group interviews (FGIs) were analysed using content analysis [28–30]. This study is based on data from children (10–14 year) and adolescents (15–18 year); both age groups will henceforth be referred to as children. In accordance with the aim, children who underwent treatment for cancer were purposefully selected to participate in this study. Thus, participants who were perceived as able to provide information to the study were invited [31]. The participants were recruited from three childhood cancer centres in Sweden: Lund, Stockholm and Umeå. In the first stage of recruitment, an email containing information about the study was sent to consulting nurses from the first author (CW). Thereafter, the consultant nurses passed on all information about the study to purposively selected families of children and asked for consent for the first author (CW) to contact them. The first author (CW) then contacted the children and their parents to provide information about the purpose of the study and participation, i.e., digital FGIs or digital individual interviews. Of 18 children contacted, 16 agreed to participate in the study. After the first contact, time proposals for the interviews, within the framework of approximately three weeks, were sent out to children and parents, where they were also encouraged to ask clarifying questions at any time via email or text messages. The children who participated had different cancer diagnoses and different lengths of disease period, they were also of different ages at diagnosis (see Table 1). All children in this study were undergoing some form of cancer treatment.

**Table 1:** Overview of characteristics

Characteristics	Children 10–18 years	
	N = 16	%
Boys	5	31
Girls	11	69
***Age when diagnosed***		
5–9 years	3	19
10–14 years	6	37.5
15–17 years	7	43.5
***How long since the diagnose***		
3–11 months	2	12.5
1–2 years	8	50
3–5 years	6	37.5
***Type of child’s diagnosis***		
Leukaemia/Lymphoma	5	31
Solid tumours	8	50
Brain tumour	3	19

### Data collection and analysis

Data collection was carried out using digital FGIs (*n* = 3, total number of participants = 10) and individual interviews (*n* = 6) during 2021–2023. In the digital FGIs the children were divided according to ages: 10–14; 10–15; 15–18 years old. Both girls and boys participated in the two FGIs with the younger children. The FGI of older children was homogeneous, consisting entirely of girls. After consent/assent from all the participants, all interviews were digitally recorded to enable transcription and data analysis. Information about the option to have the camera on or not was given, whereby one participant chose to have the camera off. The first author (CW) and last author (CB) conducted all digital FGIs and took turns moderating/taking field notes. All children were informed about that they could have their parent present during the digital FGIs. However, all children preferred not to have their parent present. The individual interviews were conducted by the first author (CW). The digital FGIs lasted between 43 and 62 min and the individual interviews lasted between 24 and 45 min. All interviews were conducted using a semi-structured interview guide (supplementary file) developed for this study. The interview guide provided a framework for keeping the discussions within the scope of the study, and for moving the discussion forward if required [31]. The semi-structured interview guide contained questions concerning the children’s views on values ​​and moral dilemmas by asking questions about self-experienced situations. Each FGI and interview began by asking participants to: *“Please tell us what is important to you when you are at the hospital and receive care.”* Participants were also asked to *“Please tell us about a situation when you did not know what the right thing to do was.”* To gain more information about the children’s views, probing questions was posed, for example, *“Could you please explain why?”* and *“How did you feel then?”* If the moderator noticed a specific child being quieter than the other children, directed questions were asked to ensure that every child had the opportunity to share their story. After each FGI/individual interview, the researcher CW or CB asked the participants if there was something more that they would like to add or ask.

After each individual interview and FGI, data were transcribed verbatim into digital word documents by the first author (CW). Inspired by Graneheim and Lundman [30], the analysis was performed in four steps: (1) The first author (CW) and last author (CB) read the transcripts carefully, reflectively and repeatedly to get an overview of what kind of values and moral dilemmas the children experienced; (2) meaning units were identified and coded by CW and CB, separately, and preliminary themes of values and moral dilemmas were identified by CW and CB, also separately; (3) a further analysis was performed by CW by identifying similarities and differences in data units, classified as themes. Thereafter all authors discussed the preliminary findings, i.e., the data in relation to the themes proposed by CW in the deeper analysis. This discussion led to a clearer structure in the analysis where some themes were reformulated and strengthened, and (4) the themes were then summarised and described in relation to the transcribed data.

### Ethical considerations

Before inviting the younger children, a dialogue with parents were conducted to find out if the child had any preferences regarding participating in digital FGIs or digital individual interviews. When the child was planned to participate in digital FGIs the question was asked if they felt alright to participate in a mixed group with girls and boys as well as different ages. All children decided independently whether they wanted to take part in the study alone or with the company of a parent. The children were informed that they could interrupt their participation at any time, without explaining why. They were also informed about that they only had to answer the questions that they wanted to answer and that it was alright to say that they did not want to answer a specific question. All paediatric oncology centres in Sweden employ psychologists or social workers who could be contacted if participation in the study caused a child to feel sad or upset.

### Findings

The findings are presented in relation to children’s values, and moral dilemmas respectively. Quotes from children are used to illustrate the themes.

### Children’s views on values

During the content analysis, five themes relating to values were found, *Personal relationships, Bodily ease and identity*, *Feeling in control and being involved, Positive Distractions*, and *Right care that is needed.*

#### Personal relationships

Almost all children described social relationships as crucial for them to feel supported. Being able to have the whole family close during the treatment was important. It was also important to keep relationships with friends as much as possible, including hanging out with boyfriends or girlfriends, both at home and in the hospital. The children emphasized that a personal relationship with HCPs was essential during their hospital stay. The approach of HCPs and their ability to be emotionally involved were described as facilitating the care situation, especially for the older children. When HCPs focused not on the illness but spoke to them as ‟friends”, they felt a special connection. Having a social and personal relationship with HCPs, as with a person outside the hospital, made them feel both emotionally safe and comfortable.

*…that you feel safe with those who work there … can talk freely with them [HCPs]… laugh and joke and all that … that you feel comfortable with those who enter your room …* Girl, age 18.

The children in this study talked about the importance of reliance on HCP’s in the relationship when communicating and providing information. Some children explained that they felt betrayed by HCPs when expectations were not fulfilled, for example, being allowed to go home, or that treatment procedures or examinations would not hurt, or would not be carried out. Some children said they protested when promises were not kept, while most children did not speak up.

#### Bodily ease and identity

According to the children in our study, bodily ease and their identity was valuable. This included that their body would not be marked by their disease, and that the disease would not affect their physical functions and abilities. ​​Having the physical capability to be with friends and to join in various activities were described as important.

*… you feel a bit like this [unfair] … in school when everyone else can be out playing and you can’t participate in certain things during the breaks …* Boy, age 11.

In almost all interviews the children described that it was important to feel well and to have symptom relief for nausea and physical pain. In various ways, children talked about how the symptoms of cancer and its treatment affected the person, i.e., their identity. It was also about how others viewed and responded to them, and for the children it was central to feel like “yourself” and being “normal” during the cancer treatment.

… *that when people … relationships that I’ve had during this time change or that people start talking to me in a different way, it makes the whole thing even worse … that then even it can’t be the same as it was before … it will be much more difficult …* Girl, age 18.

It was also valuable for the participants to see other children who were sick at the ward, as it “*made you not feel alone*” and able to identify themselves with other children affected by cancer.

#### Feeling in control and being involved

The children talked about the importance of feeling in control of their body integrity during treatment at the hospital. It was valuable for the children to feel safe and to avoid fear by being involved in the care, being well informed, and not feeling forced into procedures. In order to feel in control and involved in procedures, the children wanted to be informed about what would happen before care treatments, including the opportunity to have their say. When children’s sense of integrity was not taken into account, they felt that their body did not belong to them.

*I was about to have a [peripheral venous catheter] needle inserted [by a nurse] … then I first got a nurse who tried three times … and failed … And then another [nurse] came, and wanted to prick me, and … it didn’t work either … and then it felt a bit like they’re taking control over my body, even though … it’s me who should be able to decide over my body …* Girl, age 17.

Furthermore, it was important for the children to have various levels of involvement, including being listened to and being confirmed. Understanding and being understood meant that the sense of control was strengthened. Children, especially the older children, further described that it was important to be treated individually, with respect for their thoughts and self-determination, not only in the hospital but also when they were at home.

#### Positive distractions

Children in this study described it as meaningful to have positive distractions to kill time and to be entertained at the hospital in order to handle the fact that they could not choose to go home. They talked about the need for distractions, such as access to TV/games, clowns and play-therapy.

… *the play therapy goes around regularly … and it kind of benefits the children when I was there … I think it was good that they stopped by and checked if you want anything … maybe we should fix something … because then you can have some things to do in that … boring room …* Boy, age 13.

The opportunity to borrow books and go to the music room with friends and/or siblings was also described as important. In addition, it was valuable for the children to be at home as much as possible.

#### Right care that is needed

Children expressed the importance to get safe care, including the right care and the medical treatment that they needed to get well. To the question about what is important to you when you are treated for childhood cancer, one boy responded:

*That I get the right medicine and not the wrong one … and that I do not feel so bad … that these medicines I get so that I don’t feel bad at least make me feel a little better …* Boy, age 15.

It was also important to receive timely medication for symptom relief and their illness. Even though the children felt that all procedures and medications were uncomfortable, they talked about the importance of knowing that all procedures were for their own good.

### Children’s views on moral dilemmas

In the FGI and individual interviews children expressed several situations when they did not know what was right or how to act, due to their own value conflicts. Through content analysis three themes of moral dilemmas were identified, *Should I consider others or not?, Should I rest or not?* and *Should I refuse treatment or not?*

#### Should I consider others or not?

Considering others or not includes how much others’ behaviours or preferences should be allowed to affect the child’s well-being. Several of the children in this study had experienced an inner conflict of their values: ‘feeling in control’, versus protecting their ‘personal relationships’. This included whether it was good or not to tell HCPs that the child had been treated badly, as one 17-year-old girl said: *“… But I don’t know how to convey it* [that the doctor expressed himself badly] *to that doctor in that case … or like in what way it gets better.”* Similar moral dilemmas were about whether they should tell that they felt uncomfortable with certain HCPs and therefore did not want to be treated by them. One participant wondered if he should be more considerate of the nurse than himself. As expressed by one boy:

*… there is a nurse that I really don’t like … and I usually have her very often … It will be messy if I tell her I don’t want her. She will ask why and stuff like this and maybe get sad so I can’t take it …* Boy, age 15.

Children also talked about their uncertainty regarding their ability to make decisions on various procedural interventions such as when HCPs said there were two options for treatment. Due to a lack of knowledge, the children felt that it was impossible for them to know which treatment/care was best and caused the least harm. In these situations, they did not know who to consider the most, themselves or the HCPs.

… *like when I had my biopsy, I thought it was really scary … then they first said that I would be sedated … but that there was also an option that I wouldn’t be sedated and then I was like … I don’t know which option I want and I don’t know what is best … then there was a lot of back and forth and … I … felt that I didn’t quite decide myself but then it happened … that I had to stay awake … I guess I was just thinking what is best for the doctors … well the anaesthesiologists …* Girl, age 16.

Additionally, some children doubted whether or not they should question planned procedures which they believed to be unnecessary and irrelevant. Children also reported loyalty dilemmas in situations when both parents had different views on what was best for them (the child). The dilemma involved doubts about which parent they should listen to. For example, when one parent expressed fear of allowing bathing related to infections and the other parent said that it was ok. The child really wanted to go in the hot bathtub but at the same time did not want to worry the anxious parent. Furthermore, several children described that it was difficult to deal with others’ concerns about overexerting themselves during treatment.

#### Should I rest or not?

This theme includes how the children asked themselves whether they should save their energy or use the energy in everyday social activities. Most of the children wanted to be active and felt good about participating in leisure activities. At the same time, they described that the activity could affect their well-being negatively afterwards, due to exhaustion. According to the children, these moral dilemmas were also weighed between bad conscience and their own expectations of the body’s ability versus the body’s need for rest.

*I want to go to school to feel like normal, but on the other hand maybe I can’t bear to go to school just like usual …* Girl, age 18.

Furthermore, they compared their body’s functions and mobilization with how it was before. In almost all interviews, they described a struggle between pushing themselves to feel normal (i.e., being able to go to school, participate in training or play), or staying at home to avoid getting tired but with the risk of feeling left out.

*… that I come to a lesson or like two lessons later in the afternoon … even though they know I’m sick but, in my head, I feel like it’s going to be very anxious, and then I’ve chosen not to go to school … at all.* Girl, age 16.

They were reminded of this value conflict when parents insisted that they needed to rest or when the HCPs insisted that the body needed to be activated even when the energy was not there, due to side effects such as pain, nausea, and fatigue.

… *when I get chemo then I kind of don’t go out because I’m really tired … and moody … and I don’t want to talk to anybody … and everyone thinks I should go out … then they keep nagging at me that I have to go out and … I don’t want to because I’m really tired and can’t stand …* Girl, age 14.

#### Should I refuse treatment or not?

Children in our study described that they sometimes did not know how much physical or psychological pain they should endure when receiving the care and treatment that was best for them and that was needed. The conflicting values in the dilemma were ‘right care that is needed’, versus ‘feeling in control’. The majority of children struggled with the conflicting feelings of not wanting to undergo annoying or painful procedures, but knowing they had to in order to be cured.

… *I just started screaming and saying no, I don’t want [it] and I started crying … I hoped they would stop … but the doctor said you have to have it … it’s not a choice, you have no other choice, it’s for your sake… but I don’t want … it felt quite bad then … actually* … Girl, age 13.

One teenage female did not feel comfortable when HCPs wanted to discuss her risk of not being able to have her own children in the future and therefore wanted her to go to the reproductive centre, but she did not know what was best for her.

… *well, I could totally refuse … [to go to the reproductive centre] but I felt that I didn’t want to [refuse], I just couldn’t bear it … I had said several times that I didn’t want to … and then I thought they decided a bit … against my will …* Girl, age 17.

## Discussion

This study provides unique empirical knowledge about values ​​and moral dilemmas among children in Swedish childhood cancer care. The children in our study described a variety of values ​​that were summarized under five themes, *Personal relationships, Bodily ease and identity, Feeling in control and being involved, Positive distraction* and *Right care that is needed*. The moral dilemmas were summarized under three themes, *Should I consider others or not?, Should I rest or not?* and *Should I refuse treatment or not?*

### Discussion on children’s views on values

The themes on values can be related to what has emerged in previous research on children’s experiences of cancer treatment. For example, the importance of family and social relationships [4, 7, 32], trust in the body’s functions [33] and being included in care and get the right treatment [32]. Not unexpectedly, a trusting and *personal relationship* with the HCPs emerged as a value. Although this is in line with previous studies [4, 7, 32], one cannot ignore the children’s expressed need for personal relations with HCPs. According to Darcy and Knutsson [8], children feel lonely after receiving their diagnosis and during their treatment. In our study, the children told us that close and warm relations with HCPs facilitated the treatment experience. The feeling of ‘connection’ to HCPs might be due to a decreased feeling of alienation, and that the child’s experience of well-being is actually promoted. Further, it is possible that children’s feelings of being misled and betrayed, due to the HCPs’ dishonest information, might increase their emotions of loneliness. One can argue that having the skills to understand and act upon children’s individual needs in care is valuable, and that professional competence and continuity is closely related to fostering trusting relations. An interesting aspect is that Carlsson and Nygren [34] and Gilljam and Arvidsson [35] point out that trusting, respectful relations give the children the ability to participate in care situations. However, this, according to research findings, might be a struggle as nurses in childhood cancer care find it difficult to cultivate relationships with families [36]. This is usually related to lack of time, but also to the challenging balance between the children’s and parents’ opinions, and too often the relationship favours the parents [36, 37]. Therefore, it might be argued that the conditions and competences for building trusting relationships in childhood cancer care should be increased, in order to be able to offer and ensure good care for the family and the child. In terms of future research, it would be interesting to further explore the moral dimension of the triad of relationship and whether attention to and support in moral dilemmas might strengthen trusting relationships in childhood cancer care.

Being a growing child, in general, involves doubts and thoughts about identity, and how to be and to act. Developmentally, the age groups 10–14 and 15–18 encompass a wide range of identity building, including the clarification of personal values. During these formative years, the ongoing process of self-discovery introduces an element of uncertainty and confusion regarding one’s moral compass. Both children and adults struggle with moral dilemmas and decisions, but for children, this struggle seems to be more pronounced due to their limited life experience and stage in their life journey. Children’s existential thoughts may explain, to some extent, why participants in our study described values that involved *Bodily ease and identity.* Children with cancer probably have an additional existential layer to unravel as their life, as they knew it before their cancer diagnosis, has been disrupted [38]. In a study by Eaton Russell, Bouffet [39], children treated for childhood cancer wondered why they had cancer, what the cancerous tumour looked like, and what symptoms it caused.

According to the theme *Feeling in control and being involved*, it is relevant to discuss the complexity of children’s participation in healthcare decisions. A major responsibility rests on parents and HCPs, who must take into account the child’s need for participation in their care. Many children want to participate on their own terms, which can vary depending on both the disease situation and the mood for the day [40]. In addition, to participate in care decisions, children – like individuals in general – have unique needs. For example, some children prefer to take full responsibility [41], while others want to take selected responsibility. However, some children report that they feel neglected by not being involved [42]. For the triad of relationships involved, i.e., children, parents and HCPs, it can therefore be a balancing act to know at what level children want to be involved in decisions. Especially in cases where children’s lack of interest in participation in care clashes with the demands of parents and HCPs, the child has a right to actively participate in care, and research shows that children should be encouraged to participate [27]. These questions about how much children should be allowed to decide in childhood cancer care have also been shown to contribute to moral dilemmas among HCPs, which in some cases need to be reflected on within the care team [19]. However, our findings, as well as those of Carlsson, Nygren [34], indicate that HCPs and parents in childhood cancer care need to increase their understanding of individual children’s desires to participate in their care and treatment. More research remains to be done to understand how moral dilemmas affect the children and, specifically, if ethics support can be a tool to ease their doubts and/or meet their wishes to participate in their care. Since ethics support is currently only offered to HCPs, our goal is that it can be extended to children and parents in the near future. This could be achieved by involving children and parents in ethics rounds or providing them with individual support from ethics support staff. However, it is important that the process is evidence based and that HCPs and ethics support staff will receive specific training to reflect on children´s moral needs.

### Discussion on children’s views on moral dilemmas

A finding from our study was *Should I consider others or not*? that included the consideration the children took for others during their treatment. This was about situations where some children weighed whether it was worth telling the HCPs that their disrespectful behaviour was affecting them, or let it be. Equally, whether or not they should let the HCPs make decisions in uncertain care situations, for example treatment they did not have enough information about. In a review of end-of-life care for children, Hirata and Kobayashi [43] found that the social interactions between the triad of children, parents and HCPs were characterized by their values ​​involving, for example, caring for each other. Sometimes children’s considerations of others might be based on withheld honesty, which could negatively affect interaction in personal relations. It would be interesting to explore this further, because reliable communication was described as important to the children in our study, as well as in other research [44, 45].

The children’s inner moral conflicts about bodily energy, included under the theme *Should I rest or not*? are considered to be of an existential nature. Most of the children in this study described a desire to be seen as, and feel, normal, which is reported in other research [46]. Struggling with feelings of being “themselves” should therefore not only be linked to searching for their identity but also their health. Potential consequences for meeting their normality may be expressed in their doubts about daily activities.

The findings in this study further show that children’s moral dilemmas were about whether, and to what extent, *they should endure painful procedures or whether they should oppose the procedures*. These descriptions of moral dilemmas led us authors to wonder whether children endure situations of uncertainty, pain and discomfort because they were unsure whether they should protest. In a previous literature review in childhood cancer, it appears that HCPs often inform children that they have no choice when it comes to their care and treatment [32]. It is plausible that such information could lead to limited opportunities for children to dare to speak up. Another reason for children taking others into account and enduring painful treatments may also be due to their degree of dependence and the development of their moral reasoning. According to Miller and Harris [47], it might be the child’s cognitive development and need for self-determination that decides their ability to reason. Similarly, Kohlberg [48] states that children’s moral reasoning, from the age of eight to young teenagers, is focused on acceptance, social rules, and others’ expectations of what is appropriate or inappropriate behaviour. From this point of view, the finding that the children consider others before themselves is more understandable. Regardless, it is important to bear in mind that fear, lack of information, and uncertainty, which are common experiences among children in childhood cancer care [33], might be alleviated if the child had their say in their care [32].

### Study strengths and limitations

A strength of this research is that it is the first to focus on moral dilemmas among children between 10 and 18 years in childhood cancer care. Another strength is the triangulation during the analysis, which involved all co-authors, and enabled validation of the findings. The researchers’ different views on data increased insight and broadened the analysis [49], and helped to avoid misinterpretations and individual biases from a single researcher [50]. In addition, digital FGIs and interviews may be a strength due to ease of connecting from home, which may have contributed to less dropouts and thus research completion [51].

A possible limitation could be the technical problems encountered with digital FGIs, such as delay and interrupted audio recording due to connection problems. This sometimes led to confusion about who had the floor, which may have affected the group’s spontaneous interactions, which is what FGIs aim to achieve. Other limitations on digital FGIs could be that participation could potentially have exposed children to sensitive topics or stressful situations, which may have affected their emotional and psychological health. Parents always had the possibility to participate together with their child if the child wanted to and the researchers, trained in paediatric nursing, were attentive to any signs of stress or discomfort in the children and could terminate the session immediately if needed. However, no child exhibited such signs. Instead, there was a noticeable eagerness to share and a sense of validation from being able to express their views on their values and moral dilemmas. Furthermore, in a group setting, children might be influenced by their peers, leading them to provide responses they think are expected or accepted, rather than their true opinions. This concern was met by dividing the children according to age and thus reducing the risk of feeling pressured from an older child. The moderator´s role is very important during FGIs as they need to create a safe and comfortable environment for children to express themselves freely, which can be more challenging in a digital setting. However, conducting small groups with few participants enabled the moderators to make sure everyone had their say in a permissive atmosphere. Lastly, our findings did not include comparisons of either values ​​or dilemmas by age. It is therefore difficult to know how much the child’s cognitive maturity affected their way of perceiving both values and moral dilemmas. It may also be that the questions asked were too difficult for some of the participants to understand, which may have resulted in lack of information.

### Implications

This study provides a specific addition to other studies on children’s experiences of childhood cancer care, as it draws attention to children’s values and moral dilemmas during their treatment and increases the understanding of children’s thoughts. Furthermore, the findings in this study can be used in comparative studies on values, moral questions and dilemmas, and motivate further research on, for example, children’s participation in ethics support in order to assist them to deal with these moral challenges.

## Conclusions

This study contributes to increased understanding of children’s values and moral dilemmas in childhood cancer care. Moreover, the findings contribute to strengthening the children’s voices in care by asking about their values ​​and moral dilemmas, and not only about their psycho-social experiences. In order for children to feel safe and cared for in the hospital, HCPs need to take into account that social relationships are important and valuable to children. Children’s moral dilemmas focused mainly on questioning their own physical and psychological well-being against their expectations, the values ​​of others, and the treatment required. Further research is needed to understand how to further support the acknowledgement and handling of moral dilemmas among children when undergoing cancer treatment.

### Electronic supplementary material

Below is the link to the electronic supplementary material.

Supplementary Material 1

## Data Availability

Raw data are not publicly available to preserve individuals’ privacy under the European General Data Protection Regulation. The datasets are available from the corresponding author on reasonable request.

## Abbreviations

FGI: Focus group interview
HCP: Healthcare professional

## References

[1] National Board of Health and Welfare. National Board of Health and Welfare 2020. Stockholm, Sweden: The National Board of Health and Welfare [Socialstyrelsen].

[2] Lam CG, Howard SC, Bouffet E (2019). Science and health for all children with cancer. Science.

[3] Socialstyrelsen. National Cancer Register, https://www.socialstyrelsen.se/en/statistics-and-data/registers/national-cancer-register/ (accessed 13 august 2023).

[4] Mant J, Kirby A, Cox KJ (2019). Children’s experiences of being diagnosed with cancer at the early stages of treatment; an interpretive phenomenological analysis. Clin Child Psychol Psychiatry.

[5] Sisk B, Kang TI, Goldstein R, DuBois JM, Mack JW (2019). Decisional burden among parents of children with cancer. Cancer.

[6] Ruhe KM, Badarau DO, Brazzola P, Hengartner H, Elger BS, Wangmo T (2016). Participation in pediatric oncology: views of child and adolescent patients. Psychooncology.

[7] Nilsson S, Eriksson A, Sörman A (2021). Children’s and adolescents’ experiences of living with cancer. Nurs Child Young People.

[8] Darcy L, Knutsson S, Huus K (2014). The everyday life of the young child shortly after receiving a cancer diagnosis, from both children’s and parent’s perspectives. Cancer Nurs.

[9] Kim Y, Lee KS, Koh KN (2018). Difficulties faced by long-term childhood cancer survivors: a qualitative study. Eur J Oncol Nurs.

[10] Molewijk B, Zadelhoff Ev, Lendemeijer B, Widdershoven GAM. Implementing moral case deliberation in Dutch health care; improving moral competency of professionals and the quality of care. In Bioethica Forum 2008. pp.57–65.

[11] Rachels J, Rachels S (2019). The elements of moral philosophy.

[12] Thompson IE (2006). Nursing ethics.

[13] Johnson L-M, Church CL, Metzger M (2015). Ethics Consultation in Pediatrics: long-term experience from a Pediatric Oncology Center. Am J Bioeth.

[14] Schröder Håkansson A, Pergert P, Abrahamsson J, Stenmarker M. Balancing values and obligations when obtaining informed consent: Healthcare professionals’ experiences in Swedish paediatric oncology. Acta Paediatr. 2019. 10.1111/apa.15010 31520436

[15] Alahmad G, Al-Kamli H, Alzahrani H (2020). Ethical challenges of Pediatric Cancer Care: interviews with nurses in Saudi Arabia. Cancer Control.

[16] de Vries MC, Houtlosser M, Wit JM (2011). Ethical issues at the interface of clinical care and research practice in pediatric oncology: a narrative review of parents’ and physicians’ experiences. BMC Med Ethics.

[17] Odeniyi F, Nathanson PG, Schall TE (2017). Communication challenges of oncologists and intensivists Caring for Pediatric Oncology patients: a qualitative study. J Pain Symptom Manage.

[18] Bartholdson C, Lützén K, Blomgren K (2015). Experiences of ethical issues when caring for children with cancer. Cancer Nurs.

[19] Weiner C, Pergert P, Castor A (2022). Difficult situations and moral questions raised during moral case deliberations in Swedish childhood cancer care – A qualitative nationwide study. Eur J Oncol Nurs.

[20] Stevens PE, Pletsch PK (2002). Ethical issues of informed consent: mothers’ experiences enrolling their children in bone marrow transplantation research. Cancer Nurs.

[21] Alahmad G (2018). Informed consent in Pediatric Oncology: a systematic review of qualitative literature. Cancer Control.

[22] Weiner C, Pergert P, Castor A, Molewijk B, Bartholdson C. Sheltering in chaos: parents’ experiences when facing moral challenges in childhood cancer care. Ethics & Behavior.1–14.

[23] Quinn GP, Murphy D, Knapp C (2011). Who decides? Decision making and fertility preservation in teens with cancer: a review of the literature. J Adolesc Health.

[24] Burns KC, Hoefgen H, Strine A (2018). Fertility preservation options in pediatric and adolescent patients with cancer. Cancer.

[25] Hinds PS, Oakes L, Furman W (2001). End-of-life decision making by adolescents, parents, and healthcare providers in pediatric oncology: research to evidence-based practice guidelines. Cancer Nurs.

[26] Smith LE, Maybach AM, Feldman A (2019). Parent and child preferences and styles of Communication about Cancer diagnoses and Treatment. J Pediatr Oncol Nurs.

[27] Sweden, GOo, SFS (2018). Convention on the rights of the child.

[28] Graneheim U, Lindgren B-M, Lundman B (2017). Methodological challenges in qualitative content analysis: a discussion paper. Nurse Educ Today.

[29] Lindgren B-M, Lundman B, Graneheim U (2020). Abstraction and interpretation during the qualitative content analysis process. Int J Nurs Stud Adv.

[30] Graneheim U, Lundman B (2004). Qualitative content analysis in nursing research: concepts, procedures and measures to achieve trustworthiness. Nurse Educ Today.

[31] Polit DF, Beck CT (2017). Nursing Research. Generating and assessing evidence for nursing practice.

[32] Lin B, Gutman T, Hanson CS (2020). Communication during childhood cancer: systematic review of patient perspectives. Cancer.

[33] Comas Carbonell E, Mateo-Ortega D, Busquets-Alibés E (2021). The psychological experience of pediatric oncology patients facing life-threatening situations: a systematic review with narrative synthesis. Palliat Support Care.

[34] Carlsson IM, Nygren JM, Svedberg P (2018). Patient participation, a prerequisite for care: a grounded theory study of healthcare professionals’ perceptions of what participation means in a paediatric care context. Nurs open.

[35] Gilljam B-M, Arvidsson S, Nygren JM (2016). Promoting participation in healthcare situations for children with JIA: a grounded theory study. Int J Qual Stud Health Well-being.

[36] Ventovaara P (2023). Moral distress and ethical climate in pediatric oncology care.

[37] Mattsson J, Forsner M, Castren M (2013). Caring for children in pediatric intensive care units: an observation study focusing on nurses’ concerns. Nurs Ethics.

[38] Christensen SR, Carlsen LT (2022). From well-known to changed everyday family life in families with childhood cancer: a grounded theory of disrupted family dynamic. Psychooncology.

[39] Eaton Russell C, Bouffet E, Beaton J (2016). Balancing grief and survival: experiences of children with brain tumors and their parents. J Psychosoc Oncol.

[40] Weaver MS, Baker JN, Gattuso JS (2015). Adolescents’ preferences for treatment decisional involvement during their cancer. Cancer.

[41] Zwaanswijk M, Tates K, van Dulmen S (2007). Young patients’, parents’, and survivors’ communication preferences in paediatric oncology: results of online focus groups. BMC Pediatr.

[42] Coyne I, Amory A, Kiernan G (2014). Children’s participation in shared decision-making: children, adolescents, parents and healthcare professionals’ perspectives and experiences. Eur J Oncol Nurs.

[43] Hirata M, Kobayashi K (2023). Experiences with the end-of-life decision-making process in children with cancer, their parents, and healthcare professionals: a systematic review and meta-ethnography. J Pediatr Nurs.

[44] Souza RLA, Mutti CF, Santos RPD (2021). Hospitalization perceived by children and adolescents undergoing cancer treatment. Rev Gaucha Enferm.

[45] Jalmsell L, Lövgren M, Kreicbergs U (2016). Children with cancer share their views: tell the truth but leave room for hope. Acta Paediatr.

[46] Young K, Bowers A, Bradford N (2021). Families’ experiences of child and adolescent brain tumor: a systematic review and synthesis of qualitative research. Psychooncology.

[47] Miller VA, Harris D (2012). Measuring children’s decision-making involvement regarding chronic illness management. J Pediatr Psychol.

[48] Kohlberg L (1971). Stages of moral development. Moral Educ.

[49] Malterud K (2001). The art and science of clinical knowledge: evidence beyond measures and numbers. Lancet.

[50] Moon MD, Triangulation (2019). A method to increase validity, reliability, and Legitimation in Clinical Research. J Emerg Nurs.

[51] Halliday M, Mill D, Johnson J (2021). Let’s talk virtual! Online focus group facilitation for the modern researcher. Res Social Adm Pharm.

[52] SFS. Ethical Review Act. Stockholm: Swedish Parliament. 2008:192.

