# Supplementary material for: Children’s voices on their values and moral dilemmas when being cared and treated for cancer– a qualitative interview study
Source: BMC Med Ethics. 2024 Jun 26;25:75. doi: 10.1186/s12910-024-01075-3 (PMC11202330; doi:10.1186/s12910-024-01075-3)
Supplement: Supplementary file 1 — Supplementary Material 1 [file 12910_2024_1075_MOESM1_ESM.docx]

**Interview guide children/adolescents 10-18 years old**

We would like to talk to you about some times when you found it difficult to know what was good (and felt right) to do. We also want to talk about when something was decided about your care/treatment that you agreed or disagreed on.

.

*When you are in hospital and receive care and treatment, there are things that are and feel important.*

- **What is important to you when you receiving care?**

*There are different things that need to be decided, wherever we are (at school, at home and in the hospital).*

- **When you are in hospital receiving care, who decides then?**
- **Can you tell me/us about a time when you found it difficult to decide what you thought was the right thing to do regarding your care/treatment?**

*Sometimes you don't agree with what others think you should do (or what others think is the right thing to do).*

- **Can you tell us about a time when you disagreed with what your parents or the healthcare professionals thought?**
- **What situations lead to the most conflicts?**

*Even if we don't agree with each other, decisions often have to be made regarding care and treatment.*

- **Can you tell us about a situation when someone decided something against your will?**
- **Can you tell us about a situation when you felt unfairly treated?**
- **Is there anything else you think we need to know?**

*______________________________________________________________________*

*Possible follow-up questions for all areas of inquiry:*

- **Please tell us more…**
- **Can you provide an example?**
- **What happened? What did you do then?**
- **How did you feel then?**
- **What were you thinking then? Why do you think that is?**
